# On the Fracture Behavior of a Creep Resistant 10% Cr Steel with High Boron and Low Nitrogen Contents at Low Temperatures

**DOI:** 10.3390/ma13010003

**Published:** 2019-12-18

**Authors:** Roman Mishnev, Nadezhda Dudova, Rustam Kaibyshev, Andrey Belyakov

**Affiliations:** Laboratory of Mechanical Properties of Nanostructured Materials and Superalloys, Belgorod State University, Belgorod 308015, Russia; mishnev@bsu.edu.ru (R.M.); dudova@bsu.edu.ru (N.D.); rustam_kaibyshev@bsu.edu.ru (R.K.)

**Keywords:** martensitic steel, tensile test, bending test, fracture, brittle–ductile transition

## Abstract

An advanced, high chromium, creep-resistant steel was subjected to the tensile tests and three-point bending tests of Charpy V-notch specimens at temperatures of −196 to 20 °C. The steel exhibited ductile fracture under tension tests at all of the temperatures studied. The mechanical properties, i.e., strength and uniform elongation, were enhanced with a decrease in temperature down to −140 °C. Transgranular, dimpled fracture remained the primary fracture mechanism under tension. On the other hand, the results obtained with Charpy V-notch specimens suggested the ductile–brittle transition (DBT). Full embrittlement was observed at temperatures of −60 °C and −150 °C upon impact tests and three-point bending tests, respectively, when the unstable crack started to propagate without remarkable plastic deformation. The DBT temperature of −27 °C for the present steel corresponded to the 28 J impact transition temperature, T_28J_, when the maximum impact stress matched the maximal true tensile stress.

## 1. Introduction

High-chromium steels belonging to ferrite–martensite types are advanced materials for crucial applications in fossil power plants because of their outstanding mechanical properties at elevated temperatures [1,2]. Such steels are commonly subjected to austenitization at temperatures above approx. 1050 °C, followed by air quenching and tempering at temperatures of 750 °C to 770 °C for 2 to 4 h. The processed microstructures are characterized by a high dislocation density in martensite laths/blocks/packets and numerous second phase particles [1,2,3,4,5,6]. Among the latter ones are relatively coarse carbides of M_23_C_6_-type that precipitated mainly along the lath/block/packed boundaries, and nanoscale MX-type carbides/nitrides that homogeneously distributed throughout the tempered martensite [1,2,3,4,5]. This microstructure provides high toughness at ambient temperatures and a low ductile–brittle transition temperature (DBTT) of about −60 °C [3,5,7,8,9,10]. Moreover, these steels are characterized by a rather high impact toughness of approx. 20 J below DBTT even at −140 °C, and quite low was the 28 J impact transition temperature (*T*_28J_) of around −100 °C [7,8,9,10,11]. An aging at service temperature may decrease the impact toughness down to four times along with an increase in DBTT up to 100 °C [1,12,13,14]. Nevertheless, an impact toughness above 40 J/cm^2^ remained at ambient temperature, ensuring the reliability of steam turbine parts made of high-chromium steels [1]. Anyway, the temperature of DBT should be accurately predicted for being in order to provide the safe exploitation of the steel products.

Nowadays, a type of high-chromium martensitic steels with a high content of boron and a low content of nitrogen is considered as an advanced material for high temperature application because of its improved creep properties [4,15,16,17,18,19]. This specific alloying design results in a precipitation of M_23_C_6_ carbides along with boron-bearing M_23_(BC)_6_ particles [17,18,19,20]. On the other hand, such steels are characterized by a relatively high temperature of DBT because of numerous M_23_C_6_ precipitates that form dense arrays along the lath/block/packet boundaries and promote both the void nucleation/growth and the formation of critical cracks susceptible to rapid expansion [21]. As a result, DBTT as determined by impact tests of Charpy V-notch specimens for the 10% Cr steels, is about +10 °C, that is 73 K higher than that for P92-type steels [21].

There is an ambiguity in the use of DBTT estimated from the impact tests as a halfway point between the upper and lower shelf-absorbed energies to ensure the structural integrity of the engineering components fabricated from high-chromium steels [11]. Chatterjee et al. suggested to use the 28 J impact transition temperature as the characteristic one for the structural components [11]. High-chromium steels like P91- and P92-type steels are sufficiently tough even at temperatures below DBTT [7,8,10]. Brittle fracture was not observed on flat specimens of these steels under tension condition down to cryogenic temperatures [7]. Thus, the fracture behaviors of these steels with and without notch are quite different. Studying the fracture behavior of advanced heat-resistant steels in detail is essential to prevent the sudden failure of power plant components made of these steels at ambient temperatures during construction and/or repair.

The present work is intended to expand our previous study, which has been focused on the impact toughness of a 10% Cr steel with high boron and low nitrogen contents, to comprehensive analysis of the fracture toughness under static and dynamic loading conditions. Therefore, the primary aim of this study is to clarify the fracture behavior of an advanced 10% Cr steel, considering the stress concentrator effect and comparing the standard tensile tests of flat specimens with the bending tests of Charpy V-notch specimens. The second aim is to evaluate DBTT for static and dynamic [21] loading tests of Charpy V-notch specimens.

## 2. Experimental

The studied material was a high-chromium steel alloyed with 0.1% C, 10.0% Cr, 0.7% Mo, 0.05% Nb, 0.2% V, 0.003% N, 0.008% B, 2.0% W and 3.0% Co (in wt%). Austenitization was carried out at 1060 °C for 30 min. Then, the steels samples were air cooled followed by tempering at 770 °C for 3 h. The flat specimens with a gauge length of 35 mm and a cross-section of 7 mm × 3 mm were cut from the tempered samples and then subjected to tensile tests under isothermal conditions at different temperatures varying from −140 °C to 20 °C and at an initial strain rate of 2 × 10^−3^ s^−1^. The fracture behavior was examined by the three-point bending tests at temperatures from −196 °C to 80 °C with a crosshead speed of 2 mm/min corresponding to an initial strain rate of 2 × 10^−3^ s^−1^ using ordinary V-notch Charpy specimens. Both the tensile tests and the bending tests were carried out using an Instron 5882 (Illinois Tool Works Inc., Norwood, MA, USA) testing machine with an Instron SFL 3119-408 environmental chamber. The same holding time of 30 min at the measurement temperature was used for all samples to avoid any possible artefacts caused by variations of cooling duration [22]. The present results were compared with those obtained under dynamic loading by impact tests of standard V-notch Charpy specimens that were reported in a previous work [21].

The structural characterizations were carried out using a Jeol JEM-2100 (JEOL Ltd., Tokyo, Japan) transmission electron microscope (TEM) equipped with an INCA energy dispersive X-ray spectrometer (EDX); and the fractography studies were performed with a Quanta 200 (FEI, Hillsboro, OR, USA) scanning electron microscope (SEM). The specimens for structural observations were electro-polished using 10% perchloric acid solution in a glacial acetic acid. The transverse lath/subgrain sizes were measured on the representative TEM images by the linear intercept method counting all the clear visible boundaries and subboundaries. The dislocation densities inside the laths and/or subgrains were measured by counting the individual dislocations that observed on typical TEM images of dislocation substructures observed under multiple beam contrast conditions. The dispersed particles were studied by TEM, combining the EDX and selected area diffraction methods.

## 3. Results

### 3.1. Microstructure

**Figure 1:**
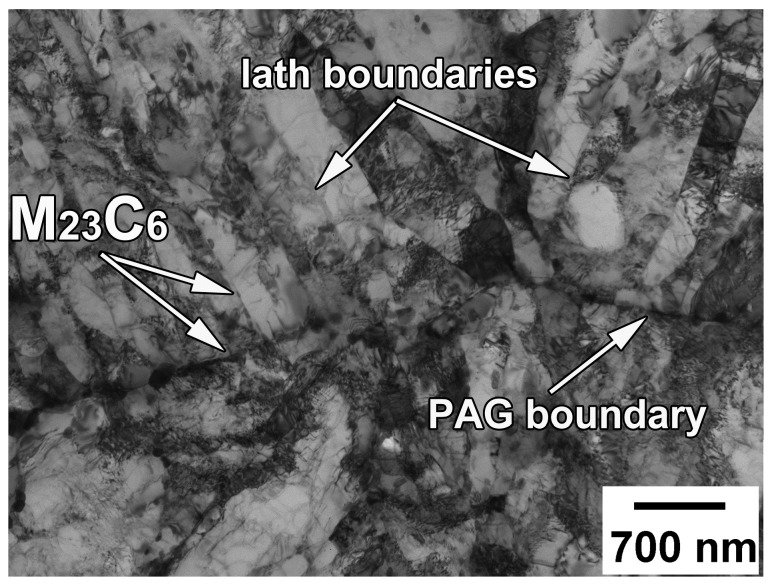
Transmission electron microscopy (TEM) micrograph of the tempered microstructure in a 10% Cr steel.

The tempered microstructure of the present steel consists of the prior austenite grains with an average size of 35 µm and the tempered laths with an average width of 0.4 µm (Figure 1). A rather high dislocation density of 1.7 × 10^14^ m^−2^ is observed inside the tempered laths. The particles of M_23_C_6_-type precipitate during tempering [4,18]. These particles with the mean size of 100 nm mainly locate on various high-angle boundaries, while those with a smaller size of about 60 nm precipitate on low-angle lath boundaries. Uniformly distributed MX-type carbonitrides are characterized by an increased content of niobium. The mean size of these particles is 30 nm.

### 3.2. Tensile Properties

**Figure 2:**
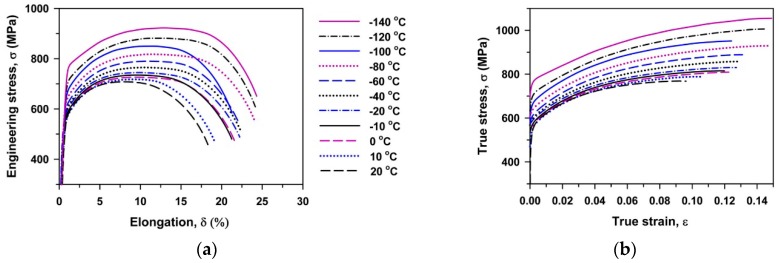
Engineering stress–elongation curves (**a**) and true stress–strain curves (**b**).

Typical engineering stress–elongation curves and corresponding true stress–strain curves that are obtained at different temperatures are shown in Figure 2. The values of yield strength (σ_0.2_), ultimate tensile strength (UTS), uniform elongation (El_u_) and total elongation (El_t_) are summarized in Table 1. Continuous yielding [7,23,24] is observed at all temperatures. The true stress–strain curves exhibit a noticeable strain hardening that provides the large uniform elongation. The maximal true stress (σ_max_) is achieved at a strain ranging from 0.06 to 0.14. It should be noted that the shape of the flow curves is almost the same irrespective of the deformation temperature. A decrease in temperature from 20 to −140 °C increases the ability of the steel to resist the plastic instability and fracture. As a result, an increase in UTS from 700 MPa to 925 MPa and an increase in total elongation from 16% to 23.5% are observed. Thus, the 10% Cr steel strengthens and becomes more ductile at cryogenic temperatures.

### 3.3. Fracture Toughness

**Figure 3:**
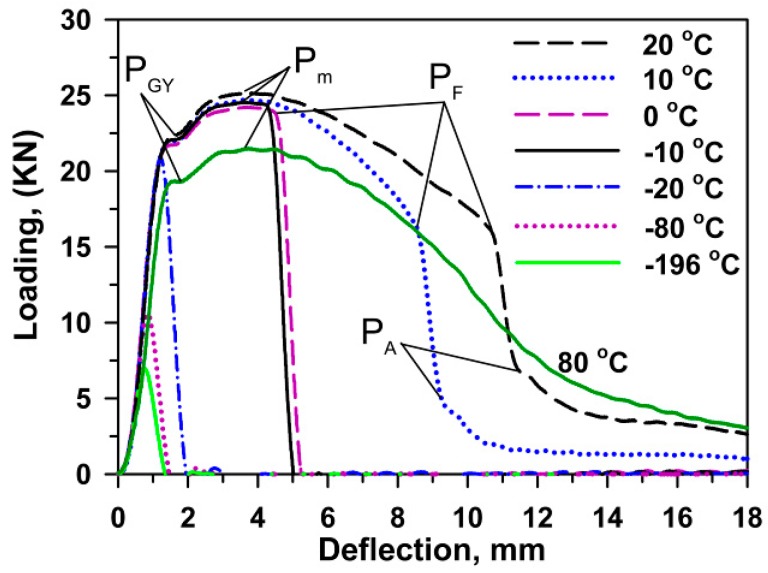
Load–deflection curves during dynamic loading. *P*_GY_ is the load at general yield, *P*_m_ is the maximum load, *P*_F_ is the cleavage fracture load and *P*_A_ is the arrest load. Reprinted from [21] with permission from Elsevier.

The impact toughness of the 10% Cr steel was studied in a previous paper [21] and can be briefly summarized here as follows. DBT corresponding to a half between the upper and lower shelf energies was observed at 10 °C. The upper shelf region with an energy of 335 J/cm^2^ is located at *T* ≥ 80 °C, and the lower shelf region is situated at *T* < −40 °C. The fracture appearance transition temperature (FATT) corresponding to the 50% of ductile surface fracture matches DBTT. The 28 J transition temperature, *T*_28J_, is about −27 °C. Typical impact load–deflection curves obtained at various temperatures are represented in Figure 3. The maximum loads, *P_m_*, the general yield loads, *P_GY_*, the well-defined specific loads, *P_F_*, corresponding to the unstable crack propagation, and the loads for the fracture arrest, *P_A_*, [12,25] are marked in Figure 3.

**Figure 4:**
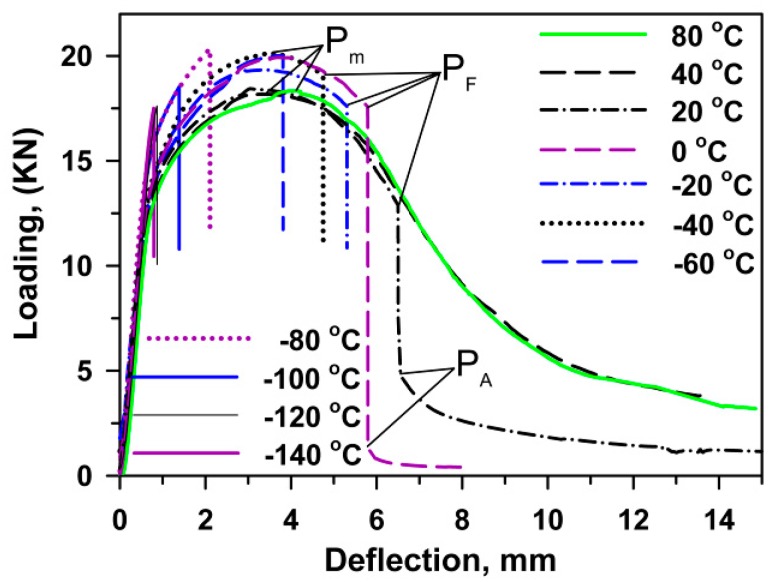
Load–deflection curves obtained during the three-point bending tests at different temperatures. *P*_m_ is the maximum load, *P*_F_ is the cleavage fracture load, *P*_A_ is the arrest load.

Figure 4 shows typical load–deflection curves obtained during the three-point bending tests. It is seen that the fracture occurs in a viscous manner at temperatures of *T* ≥ 40 °C. The maximum load is attained after a relatively large deflection of around 4 mm, which is a half of the transverse size of the central specimen section. Sharp *P*_F_ corresponding to the onset of unstable cleavage can be recognized at temperatures of *T* ≤ 20 °C. An arrest load and total deflection decrease with temperature.

**Figure 5:**
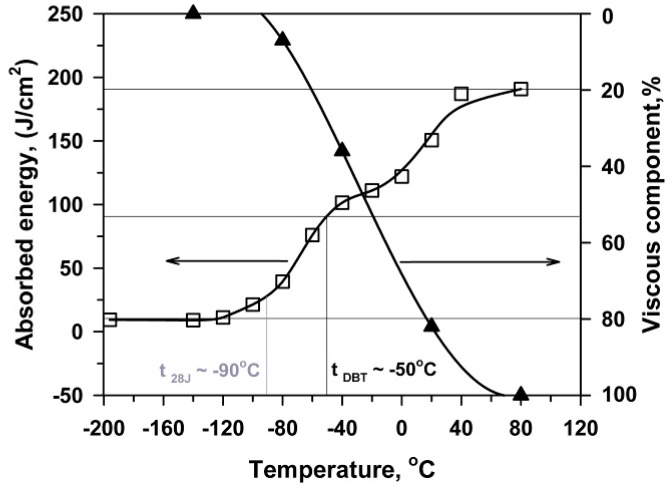
Effect of temperature on the V-notch Charpy absorbed energy during the three-point bending tests and the shear fracture percentage.

The fracture toughness curve obtained from the three-point bending tests and the fraction of shear fracture are shown in Figure 5. The upper shelf region of Charpy V-notch energy is observed at *T* ≥ 50 °C. The transition region is observed in the temperature interval of −120 to 20 °C. At *T* < −120 °C, the steel is fully brittle with a low absorbed energy of 9 J/cm^2^, which remains unchanged down to liquid nitrogen temperature. At *T* = −50 °C corresponding to a halfway point between the upper and lower shelf energies in Figure 5, which can be considered as DBTT for the three-point bending tests, the Charpy V-notch energy is about 100 J/cm^2^. The values of FATT and *T*_28J_ are −22 °C and −90 °C, respectively. Therefore, almost full brittle fracture occurs during the three-point bending tests at *T* < −90 °C.

The transition from the upper to lower shelf region in Figure 5 is accompanied with a gradual increase in *P*_m_ (Figure 4). At *T* > DBTT, unstable crack propagation follows a stage of stable crack propagation. The onset of unstable crack propagation appears at *T* < DBTT just after the maximum load is reached. At the lower shelf region, the load at which the unstable crack propagation appears decreases with decreasing temperature and a very small deflection is observed. Hence, the high fracture toughness during the three-point bending tests is attributed to a high displacement of the pendulum required for attaining the maximum load followed by stable crack propagation. The suppression of stable crack propagation corresponds to DBTT. The premature of unstable cleavage at the hardening stage occurs in the temperatures below DBTT.

### 3.4. Fractography

#### 3.4.1. Tension Tests

**Figure 6:**
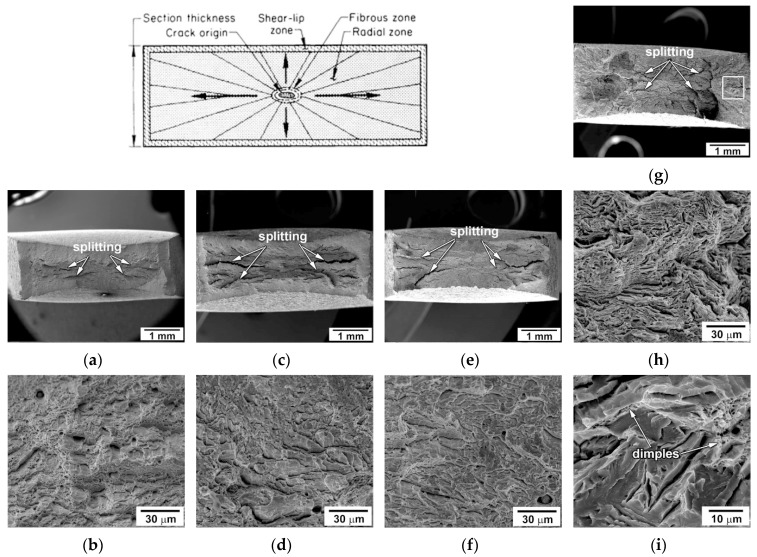
Scanning electron microscopy (SEM) micrographs showing the fracture surfaces of the specimens tensioned at 20 °C (**a**,**b**), −40 °C (**c**,**d**), −60 °C (**e**,**f**), and −140 °C (**g**,**h**,**i**) along with a scheme of the fracture surface markings showing specific areas of rectangular tensile specimen.

Figure 6 represents the overall views of the fracture surfaces of tensioned specimens. The transgranular mode is the main fracture mechanism at all temperatures. Three well-known fracture signatures, i.e., fibrous zone, radial zone and shear-lip zone [26,27], can be distinguished in Figure 6. The dimension of these zones depends upon temperature. It is known [26,27] that the triaxial stresses in the necked section develop the highest shear stress at the center of a tensile specimen forming a central crack. Next, this crack grows slowly, forming the fibrous zone of ductile fracture with an elliptical shape [26,27]. At ambient temperature, a large fibrous zone consists of fine and deep dimples (Figure 6a,b), indicating the ductile character of subcritical crack growth that requires high energy. The transition to shallow dimples and increasing dimple dimensions occurs with decreasing temperature. The size of the fibrous zone tends to decrease with a decrease in temperature. An evidence for the intergranular fracture can be found at cryogenic temperatures. The canyons with slip features on walls are observed along the boundaries of prior austenite grains at −140 °C. However, the role of this fracture mechanism is minor.

The relief of the radial zone with the coarse radial marks, secondary cracking and crack branching is indicative of increasing the crack growth rate. The dimple fracture and the coarse curved radial marks in the radial zones support this conclusion. A decrease in the temperature leads to an expansion of the radial zone, increases the number of secondary cracks and promotes crack branching. The shear-lip zone of quasi-cleavage fracture can be distinguished at *T* ≥ −40 °C.

This zone is apparently one of unstable crack propagation. The size of the shear-lip zone decreases as temperature decreases. At −140 °C, the formation of an unstable crack front requires high energy. At this temperature, the unstable crack propagation occurs in a quasi-cleavage manner, while the dimples can only be found on a few tear ridges.

#### 3.4.2. Three-point Bending Tests

**Figure 7:**
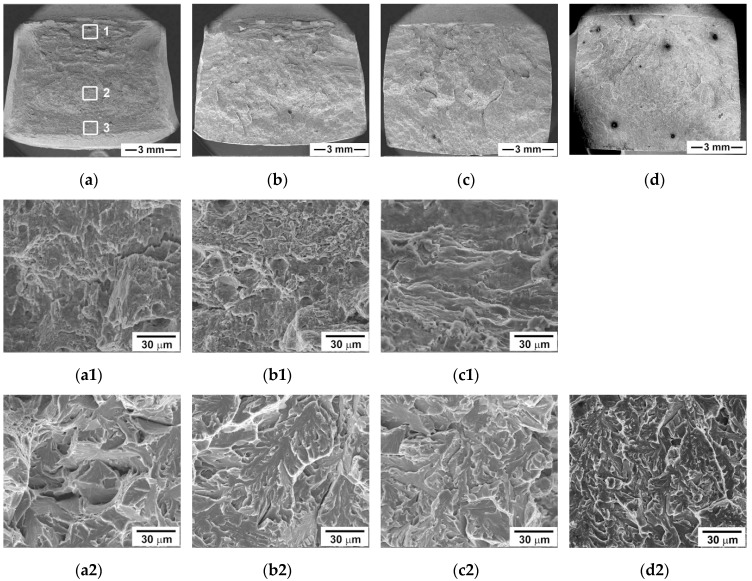

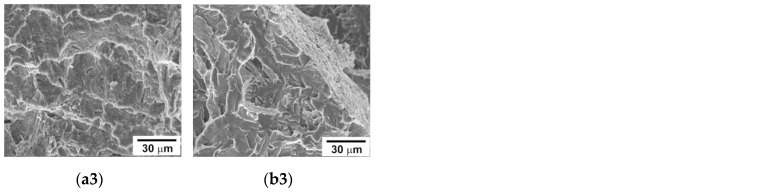
Macrographs of the fracture surface in the Charpy V-notch specimen after three-point bending test at 20 °C (**a**), −40 °C (**b**), −80 °C (**c**)and −140 °C (**d**) along with the SEM images of the crack initiation/stable crack propagation zone (portion 1), the unstable crack propagation zone (center portion 2) and the shear-lip zone (portion 3) in low to high magnifications.

The fracture surfaces of the V-notch Charpy specimen after bending tests with the low strain rate are quite different from those after tensile tests (Figure 7). The fracture surface of the specimen tested at room temperature consists of a general sequence of all characteristic fracture zones, i.e., the zone of fracture initiation followed by the fibrous zone of stable crack propagation, then the zone of unstable crack propagation, and the final shear-lip zone of arrested crack propagation (Figure 7a). The zone of unstable crack propagation covers the largest area of the fracture surface irrespective of test temperature. Decreasing temperature expands this unstable zone and reduces the other zones until their complete disappearance. The shear-lip zone almost disappears at *T* < 0 °C (Figure 7c,d). This correlates with an absence of the arrest of crack propagation on the load-deflection curves in Figure 4.

At ambient temperature, the stable crack propagation in the initiation and fibrous zones occurs through the ductile mechanism. The transition to unstable crack propagation is attributed to the transition from dimple fracture to quasi-cleavage fracture mode in the fibrous zone. In addition, the intergranular fracture along the grain boundaries is observed in the unstable crack propagation zone (Figure 7a). The voids can nucleate on the boundaries of prior austenite grains. However, the propagation of crack fronts along these boundaries occurs in a few grains. The transgranular cracking is dominant. Cleavage fracture is arrested in the shear-lip zone, where the coarse and fine shallow dimples suggest ductile fracture. At −40 °C, the fine dimples are dominant in the initiation and fibrous zones (Figure 7b). The quasi-cleavage fracture occurs in the shear-lip zone, where very fine dimples are observed on the extended tear ridges. The area of stable crack propagation reduces with decreasing the temperature (Table 2). The unstable crack propagation occurs after the formation of a subcritical crack front in the initiation zone. Therefore, the previously created flaw significantly modifies the fracture mechanisms. At *T* > *T*_28J_, the unstable cleavage fracture occurs after the stages of subcritical crack initiation and stable crack propagation. In contrast, V-notch provides higher local stresses than required for unstable cleavage fracture at *T* < *T*_28J_. Under such conditions, V-notch plays a role of a crack susceptible to unstable propagation.

## 4. Discussion

**Figure 8:**
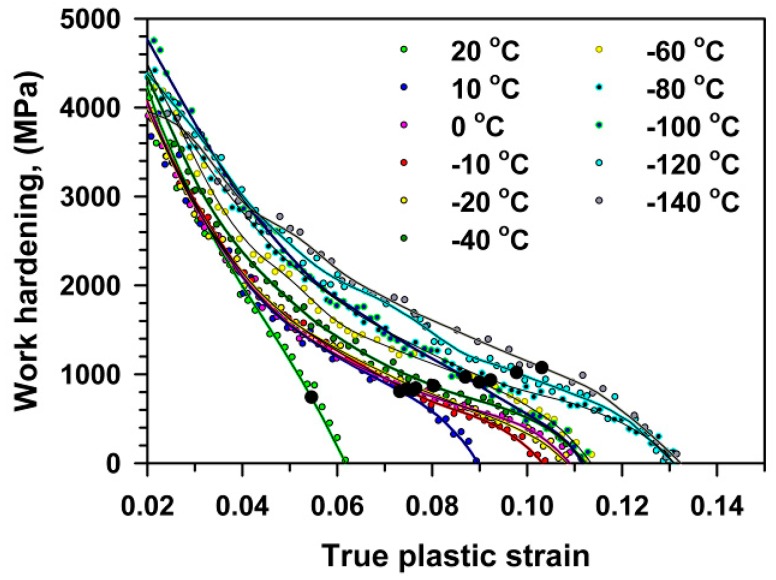
Strain-hardening during tensile tests at different temperatures.

**Figure 9:**
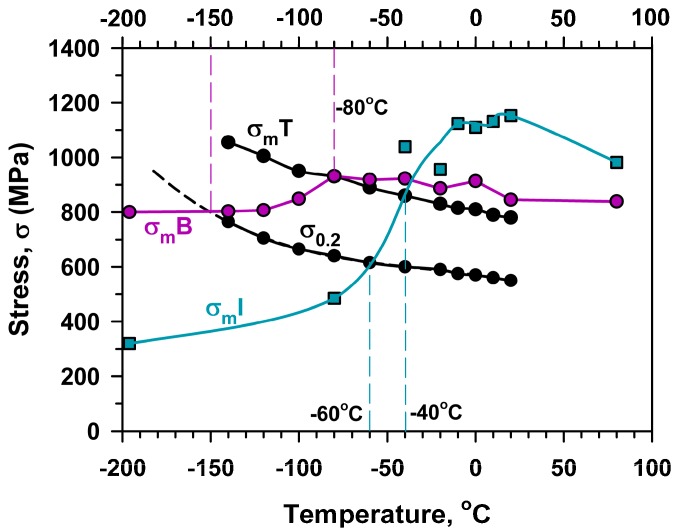
Temperature dependence of the maximum stresses during impact (σ_m_^I^) and three-point bending (σ_m_^B^) tests calculated by Equation (1) along with the yield strength (σ_0.2_) and the maximal stresses (σ_m_^T^) during tensile tests.

High-chromium steels are a unique material that exhibits a concurrent increase in strength and ductility with decreasing temperature [7]. Uniform elongation increases with a decrease in tensile temperature that is usually related to the increased stability of plastic flow [7,24,28,29]. This presumption is supported by the analysis of the strain-hardening, *d*σ/*d*ε, calculated as the first derivative of the true stress from the true strain (Figure 8). The strain-hardening during tensile tests can be subdivided into three stages. Those are the first stage of a rapid decrease in *d*σ/*d*ε at early deformation, the second stage of parabolic strain dependence of *d*σ/*d*ε and the third stage of accelerated decrease in *d*σ/*d*ε. It is clearly seen in Figure 9 that the magnitude of *d*σ/*d*ε increases as temperature decreases. The value of *d*σ/*d*ε corresponding to the second stage onset increases from 2000 to 2800 MPa with a decrease in temperature from 20 °C to −140 °C. This enhances both uniform elongation and total elongation-to-failure. It is worth noting that P92-type steels exhibit the same work hardening behavior at cryogenic temperatures despite the dominance of quasi-cleavage fracture in the tensioned specimens [7]. The unique temperature effect on the strength and ductility in high-chromium steels is similar to that for fccalloys [7,28]. Therefore, these steels are highly tough in tension down to liquid nitrogen temperature in the absence of a notch, because a high energy is necessary for the formation of critical macrocracks susceptible for unstable propagation.

Let us consider the notch effect on the fracture behavior in more detail. The maximal tensile stress in the minimum cross section of the specimen at the notch root can be evaluated as follows [25]:

(1) $$\sigma =\frac{\beta LP}{2C_{f}{\left(W-a\right)}^{2}B}$$

where *L* is the span (40 mm); *P* is the load; *W* is the specimen width (10 mm); *B* is the specimen thickness (10 mm); *a* is the notch depth (2 mm); *C*_f_ is the constraint factor (the ratio of the yield load to the load required to yield an unnotched specimen) that depends on the instrument tip (*C*_f_ = 1.363 for ASTM tip [7,25]); and *β* is a constant depending on the yielding criterion (it is equal to 2 for the Tresca criterion [7,25]).

**Figure 10:**
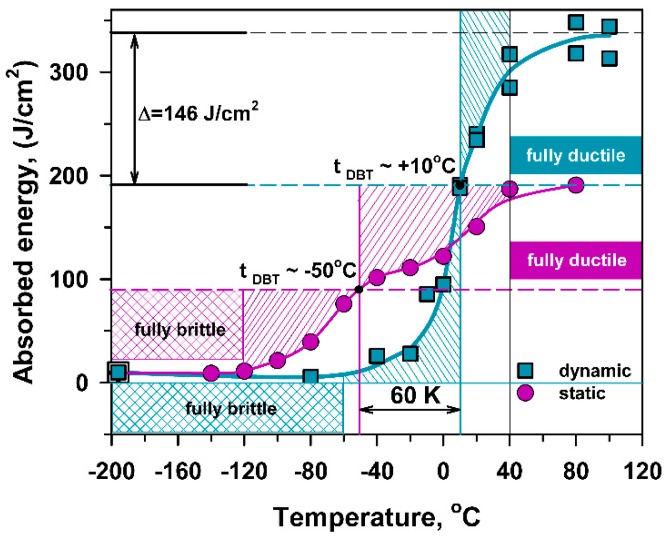
Effect of temperature on the V-notch Charpy absorbed energy for impact [21] and three-point bending tests corresponding to dynamic and static loading.

The maximum stress and the brittle fracture stress for impact and three-point bending tests, σ_m_^I^, σ_F_^I^, σ_m_^B^, σ_F_^B^, respectively, calculated by Equation (1) using the values of maximal (P_m_) and fracture (P_F_) loads (Figure 3 and Figure 4), are presented in Figure 9 along with σ_0.2_ and σ_m_^T^ obtained from tensile tests (Figure 2, Table 1). An inspection of Figure 9 with the temperature dependence of the V-notch Charpy-absorbed energy during impact and the three-point bending tests (Figure 10) suggests that the already created flaw plays a key role in the DBT of the high-chromium steel. Charpy V-notched tests under the static and dynamic loading show evidences for rather large plastic deformation providing significant absorbed energy at *T* ≥ *T*_28J_ [23,25,30]. At *T* < *T*_28J_, the steel becomes brittle, and the already created flaw initiates an unstable crack propagation with a quite small zone of plastic deformation ahead of the flaw [7,23]. In the upper shelf region, the dynamic loading provides an almost 75% increase in the Charpy V-notch absorbed energy compared with the static loading (Figure 10). This is associated with higher stresses for crack initiation and the larger stage of stable crack propagation during impact tests than during three-point bending tests (Figure 3 and Figure 4).

According to the Yoffee diagram [31,32,33,34], the brittle fracture should occur at temperatures below an intersection of the σ_0.2_ and σ_m_ curves for different types of loading, i.e., σ_m_^I^ and σ_m_^B^ for impact and three-point bending tests, respectively (Figure 9). Under dynamic loading, the temperature of the σ_0.2_ and σ_m_^I^ intersection is higher than that for the static loading (three-point bending tests). These characteristic temperatures of −150 °C and −60 °C for the static and dynamic loading, respectively, can be considered as the temperatures of embrittlement. This is also confirmed by the fully brittle fracture and the low absorbed energy in the temperature range corresponding to σ_0.2_ ≥ σ_m_.

The effect of a stress concentrator on the fracture behavior can be clarified by a comparison of σ_m_^T^ with σ_m_^I^ and σ_m_^B^ in Figure 9. The maximal true stresses correspond to the onset of necking during tensile tests, and the maximal impact/bending stress is indicative for the crack formation and propagation during V-notch tests [7,21]. The stresses required for the neck formation (σ_m_^T^) during the tensile tests and those for the crack initiation (σ_m_^B^) during the three-point bending tests are the same in the range of *T* ≥ −80 °C because of almost the same strain rates in these two types of loading. Therefore, the presence of V-notch does not affect the fracture at *T* ≥ −80 °C, whereas at lower temperatures the stresses required to fracture initiation for the notched specimens are much lower than the maximal true stresses during tensile tests. On the other hand, the maximal impact stress attains σ_m_^T^ at *T* = −40 °C. The values of σ_m_^I^ exceed σ_m_^T^ at *T* > −40 °C. This is attributed to the significant absorbed energy at the stage of crack initiation. At *T* < −40 °C, σ_m_^I^ is lower than σ_m_^T^ because the energy for crack nucleation during impact loading of V-notched specimens rapidly decreases with temperature. Thus, the ability of the steel to resist the formation of a crack with critical dimensions is a key factor to prevent fracture upon dynamic loading. It is obvious that *T*_28J_ = −27 °C adequately describes DBT under dynamic loading. At this temperature, the true stress for the onset of necking during tension and the stress for crack initiation are nearly the same (Figure 9). Therefore, *T*_28J_ determined by the impact test could be considered as a critical temperature describing DBT for the ductile high-Cr steels, instead of DBTT measured as that corresponding to the half between the upper shelf and lower shelf energies.

The high work-hardening rate during tensile tests provides the high stored energy before necking. In addition, the formation of a crack with a critical size results from the propagation of microcracks in a ductile manner. Hence, a high energy is necessary to initiate the unstable crack propagation [7], and the steel is tough even at cryogenic temperatures. An embrittlement is a phenomenon of the notched specimens of this steel; it appears at temperatures when the size of the previously created flaw becomes higher than the size of the critical crack susceptible to unstable propagation. If the size of the previously created flaw is lower than the dimension of the critical crack, the stable crack propagation takes place and the steel is tough. The toughness of the steel is controlled by the area of stable crack propagation.

## 5. Conclusions

The fracture behavior of a 10% Cr steel with 0.003% N and 0.008% B was studied in the tensile tests and three-point bending tests of Charpy V-notch specimens. The main results can be summarized as follows.

1. Total elongation and ultimate tensile strength increased from 18% to 24% and from 710 MPa to 925 MPa, respectively, with a decrease in temperature from 20 °C to −140 °C due to an expansion of the strain hardening stage. The formation of crack with critical dimension occurred upon necking accompanied by microcrack nucleation and growth in a ductile manner absorbing high energy. The unstable crack propagation in a brittle manner was observed at −140 °C only.
2. A temperature of −50 °C corresponded to the half of the sum of the upper shelf energy and the lower shelf energy, and was considered as a temperature of ductile–brittle transition for three-point bending tests of V-notched specimens. This temperature was lower than the fracture appearance transition temperature by about 30 °C. A rather low 28 J transition temperature, *T*_28J_ = −90 °C, was attributed to short-range crack propagation in stable manner. The maximal stress during three-point bending and the maximal true stress during tensile tests were almost the same at temperatures above −80 °C. In contrast, the stresses required to fracture initiation for the notched specimens were much lower than the maximal true stresses during tensile tests at *T* < −80 °C. Therefore, the presence of V-notch scarcely affects the fracture at *T* ≥ −80 °C, whereas the steel becomes highly susceptible to flaw lower temperatures.

## Figures and Tables

**Table 1:** Yield strength (σ_0.2_), ultimate tensile strength (UTS), maximal true stress (σ_m_^T^), uniform elongation (El_u_), and total elongation (El_t_) of a 10% Cr steel at different temperatures.

Parameters	Temperature, °C										
	20	10	0	−10	−20	−40	−60	−80	−100	−120	−140
σ_0.2_, MPa	550	560	570	575	590	600	615	640	665	705	765
UTS, MPa	710	720	725	735	745	765	790	820	850	880	925
σ_m_^T^, MPa	780	790	810	815	830	860	890	930	950	1005	1055
El_u_, %	7.2	8.9	9.1	7.7	9.7	8.8	10	10.8	10.5	10	12.1
El_t_, %	17.7	18.5	21	20.6	21.6	21.8	21.5	23.4	20.5	23.4	23.5

**Table 2:** The zone sizes (mm) as measured on SEM images crosswise to the notch.

Zone	Test Temperature, °C				
	80	20	−40	−80	−140
Initiation and fibrous zones	4.3	2.8	1.5	0.2	0.1
Unstable crack zone	-	2.9	5.5	7.7	7.9
Shear-lip zone	3.5	3.3	0.7	0.1	-
Total	7.8	7.9	7.7	8.0	8.0
